# The outcomes and controversies of transplant tourism—Lessons of an 11-year retrospective cohort study from Taiwan

**DOI:** 10.1371/journal.pone.0178569

**Published:** 2017-06-02

**Authors:** Daniel Fu-Chang Tsai, Shi-Wei Huang, Soren Holm, Yi-Ping Lin, Yu-Kang Chang, Chih-Cheng Hsu

**Affiliations:** 1 Graduate Institute of Medical Education and Bioethics, National Taiwan University College of Medicine; Department of Medical Research, National Taiwan University Hospital; and Centre of Biomedical Ethics, National Taiwan University, Taipei, Taiwan; 2 Department of Urology, National Taiwan University Hospital Yunlin Branch, Yunlin, Taiwan; 3 School of Law, University of Manchester, Manchester, United Kingdom; 4 Office of Health Care Policy Research, Koo Foundation Sun Yat-Sen Cancer Center, Taipei, Taiwan; 5 Institute of Population Health Sciences, National Health Research Institutes, Zhunan, Taiwan; 6 Department of Medical Research, Tungs’ Taichung Metro Harbor Hospital, Taichung, Taiwan; 7 Department of Health Services Administration, China Medical University, Taichung, Taiwan; University of Toledo, UNITED STATES

## Abstract

**Background:**

Transplant tourism has increased rapidly in the past two decades, accounting for about 10% of world organ transplants. However it is ethically controversial and discouraged by professional guidelines. We conducted this study to investigate the outcomes and trends of overseas kidney and liver transplantation in Taiwan to provide a sound basis for ethical reflection.

**Methods and findings:**

The Taiwanese National Health Insurance Research Database was used to identify 2381 domestic and 2518 overseas kidney transplant (KT) recipients from 1998 to 2009 and 1758 domestic and 540 overseas liver transplantation (LT) recipients from 1999 to 2009. Cox proportional hazards models were used to assess the risks of mortality and graft failure. The numbers of overseas transplantation increased after 2000, reached a peak in 2005 and decreased after 2007. Compared to their domestic counterparts, the overseas KT recipients were older, male predominant, with shorter pre-op dialysis period and more comorbidities. Similarly, the overseas LT recipients were older, male predominant and had more hepatocellular carcinoma cases. The 1-, 5-, and 10-year patient survival rates were 96.9%, 91.7% and 83.0% respectively for domestic KT and 95.8%, 87.8% and 73.1% for overseas KT (p<0.001). The 1-, 5-, and 10-year patient survival rates were 89.2%, 79.5%, 75.2% for domestic LT and 79.8%, 54.7%, 49.9% for overseas LT (p<0.001).

**Conclusion:**

The poorer outcomes of the overseas groups may be due to more older patients, more comorbidities (KT), or more hepatocellular carcinoma recurrences (LT). After domestic reform and international ethical challenges, the numbers of organ tourism decreased but the practice still persisted surreptitiously. Compulsory registration policies for overseas transplantation with international conventions to sanction organ trafficking and transplant tourism should be considered to stop these controversial practices.

## Introduction

The advancement of organ transplantation has saved numerous human lives and created enormous welfare gains. However, in the past two decades, the global organ shortage has led to the development of transplant tourism: the practice of traveling outside one’s own country to obtain organ transplantation, which often involves organ trade or trafficking [1]. Transplant tourism was estimated to account for 10% of organ transplants performed around the world in 2007 [2]. Such practice, though saving lives, has been discouraged by many international organizations because it involves the exploitation of vulnerable groups and the poor [1,3].

Taiwan’s hepatitis B carrier rate and the country’s prevalence and incidence of renal dialysis are among the world’s highest [4,5]. Even though Taiwan was one of the first Asian countries to perform renal transplant surgery in 1968, it has suffered from a severe shortage of transplantable organs for more than four decades due to a low organ donation rate. As a result, transplant tourism from Taiwan to China began in the early 1990s and has progressed rapidly as social and economic interaction between the two countries have increased [6,7]. According to a survey in 2006 by Taiwan’s Department of Health, only 2 of 400 overseas kidney transplant (KT) recipients and 3 of 222 overseas liver transplant (LT) recipients had organ transplantation performed outside of China [8]. Due to a growing awareness of the ethical controversies and human rights issues, measures were taken to discourage transplant tourism. For example, in 2006, the Taiwanese government announced a guideline prohibiting doctors’ participation in any form of organ brokering [9], and in 2007, requested physicians’ voluntary reporting of overseas transplant patient information to the Taiwan Organ Registry and Sharing Center (TORSC). Meanwhile, China introduced its Human Organ Transplant Act in 2007 [10]; and in 2008, the Declaration of Istanbul prohibited transplant tourism [1].

However, the practices and the outcomes of international organ tourism have not been well understood. Nationally-integrated and comprehensive medical and social research concerning transplant tourism is still scant. Questions to be answered include “How are patients who engaged in transplant tourism different from other patients?” and “Are there differences in the outcomes for overseas and domestic transplants?” Since Taiwan’s National Health Insurance (NHI) is a compulsory and universal health insurance program that covers over 99% of the general population and keeps comprehensive healthcare records, an overview of the overseas transplant patient population and the outcomes of the transplants are available [11]. Therefore, we investigated trends over the past decade in the numbers and outcomes of overseas kidney and liver transplants and in transplant-related policies in order to create an evidence base for reflection upon ethical/legal implications leading to specific proposals for policy initiatives in the Asian region that may help to resolve relevant important global health, ethics, and human rights issues.

## Subjects and methods

### Data source

All patients in Taiwan who need organ transplantation and/or post-transplantation immunosuppressive therapies are registered in the NHI program so that their costs of treatment can be covered. Therefore, all transplant recipients (both domestic and overseas) in Taiwan can be identified from the NHI Research Database (NHIRD), which is derived from NHI reimbursement claims since 1996.

### Study subjects

Those who received KT or LT were divided into two groups: “domestic recipients” (Taiwanese receiving a transplant in Taiwan) and “overseas recipients” (Taiwanese receiving a transplant abroad). From the NHIRD, we identified 2381 domestic KT via NHI records for the KT procedure between January 1998 and June 2009. A total of 68 transplants were excluded because of a second KT or with a simultaneous LT. To make domestic and overseas KT comparable, we further excluded 63 subjects who died or resumed dialysis within 1 month after the domestic KT operation, because only the successful overseas transplantation patients who returned to Taiwan and received anti-rejection therapies could be included in our study. Therefore, the remaining 2250 domestic KT recipients were selected for further analysis.

We defined overseas KT recipients as patients who were prescribed immunosuppressive medication by Taiwan physicians for kidney transplants (ICD9 = V42.0) but did not have an NHI record for a KT operation. The overseas KT recipients were validated with the NHI-based registry of catastrophic illness to exempt co-payment, and transplantation, cancer and dialysis were all included in the designated categories of catastrophic illness. The transplant-related immunosuppressive drugs recognized in this study include cyclosporine, tacrolimus, mycophenolate mofetil, sirolimus, rapamune, and cytotect. Among the 2518 overseas KT identified between January 1998 and June 2009, 114 transplants were excluded because of a second KT or with a simultaneous LT. The remaining 2404 overseas KT recipients were selected for further analysis. By applying similar criteria in selecting domestic and overseas LT recipients from the NHIRD, we identified 1658 domestic LT recipients (excluding 84 patients who died within one month after LT and 16 secondary LT) and 540 overseas LT recipients for further analysis between January 1999 and December 2009. We further contacted the TORSC to get the number of overseas and domestic (including deceased and living) transplants beyond the study period.

### Statistical analysis

The distributions of demographic and clinical characteristics of the study subjects were described and compared using mean ± standard deviation (SD) and Student’s t-tests for continuous variables, and counts/proportions and chi-square tests for categorical variables. The comorbidity was measured by the D’Hoore’s Charlson comorbidity index (CCI) score [12] using the subjects’ NHI records a year prior to transplantation. The trends of overseas and domestic transplants were compared using Cochran-Armitage trend test.

Associations between domestic and overseas KT recipients and mortality/graft failure (or association between LT groups and mortality) were analyzed using Kaplan–Meier survival curves and log-rank tests. Multivariable Cox proportional hazards models were further conducted to estimate their adjusted associations. The proportional hazards assumption was evaluated by plotting Kaplan–Meier survival curves for investigated covariates against follow-up time. Study entry was defined as the date of transplantation. For domestic KT and LT recipients, the date of transplantation was as shown in the NHIRD. For overseas KT and LT recipients, the date of transplantation was defined as 14 days and 35 days, respectively, prior to the date the patients took the first prescription of post-transplant immunosuppressant drugs, because the average postoperative hospital stays for overseas KT and LT were 14 days and 35 days, respectively, according to a previous questionnaire survey (8). As determined by database availability, the KT and LT cohorts were followed up through the ends of 2009 and 2010, respectively. In the models estimating the hazard ratio (HR) of mortality, observations were censored on December 31, 2009, for kidney transplants and December 31, 2010, for liver transplants, or on the date that the patients died, whichever occurred first. In the models estimating the hazard ratio of kidney graft failure, observations were censored on December 31, 2009, on the date that the patients died, or the date on which the subjects resumed persistent dialysis, whichever came first. The pre-transplant characteristics adjusted in the multivariable Cox proportional hazards models for KT recipients included gender, age at KT, CCI score, and time interval between initiation of dialysis and KT. To assess mortality risk for LT recipients, age, gender, CCI score, and hepatocellular carcinoma, were adjusted in the multivariable models.

Analyses were performed using SAS software, version 9.3 (SAS Institute, Cary, North Carolina, USA). A two-sided P value < 0.05 was considered statistically significant. This study was approved by the Institutional Review Board of the National Health Research Institutes.

## Results

### Numbers of transplants and trends

**Fig 1:**
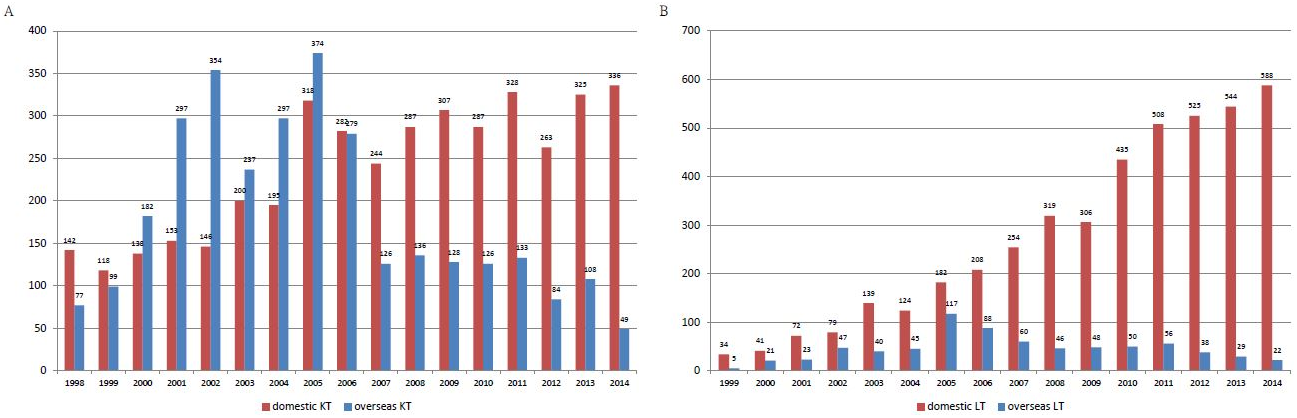
Number of domestic vs overseas transplants, 1998–2014. (A) Kidney transplants, 1998–2014 (Cochran-Armitage trend test P < 0.0001). (B) Liver transplants, 1999–2014 (Cochran-Armitage trend test P < 0.0001). KT = kidney transplant. LT = liver transplant. The numbers from 1998–2009 and 2010–2014 were obtained from NHIRD and TORSC, respectively. The numbers in 2009 would be incomplete since some recipient data were not available until in 2010 NHIRD.

The number of patients receiving KT overseas has increased since 2000 and first peaked in 2002 (n = 354) before a decrease in 2003 (Fig 1), the year of the severe acute respiratory syndrome (SARS) epidemic in Southeast Asia [13]. After a second peak in 2005 (n = 374), cases of overseas KT decreased in 2007–2014 (P<0.001). Meanwhile, the number of overseas LT started to increase in 2000, peaking in 2005 as well (n = 117), and then decreased (P<0.001). The steady decrease of overseas LT after 2012 coincided with an increase of domestic LT, which was mainly from related living donations (S1 Table).

### Demographic comparison

Table 1 shows that, compared to the domestic KT recipients, the overseas KT recipients were older (47.9 vs 41.2 years, p<0.001), male-dominant (54.8% vs 49.0%, p<0.001), and had a shorter dialysis duration before KT (p<0.001). The overseas KT recipients also suffered from more comorbidities: they had a higher CCI score (1.01 vs 0.73, p<0.001), as well as higher percentages of diabetes (14.2% vs 8.0%, p<0.001) and hypertension (38.6% vs 26.5%, p<0.001).

**Table 1:** Characteristics of kidney transplant recipients from January 1, 1998 to June 30, 2009, domestic vs overseas.

	Domestic KT recipients (N = 2250)		Overseas KT recipients (N = 2404)		*p*-value*
	n	%	n	%	
Sex (male)	1103	49.0	1318	54.8	<0.001
Age at KT (years, mean ± SD)	41.2 (12.2)		47.9 (11.5)		<0.001
≤35	666	29.6	336	14.0	<0.001
35–45	648	28.8	551	22.9	
45–55	673	29.9	834	34.7	
55–65	230	10.2	545	22.7	
>65	33	1.5	138	5.7	
Duration of dialysis before transplantation					
0	193	8.6	316	13.2	<0.001
<1 year	647	28.8	974	40.5	
≥1 year	1410	62.7	1114	46.3	
CCI score^†^ (mean ± SD)	0.73 (1.19)		1.01 (1.38)		<0.001
0	1374	61.1	1212	50.4	<0.001
1–2	682	30.3	863	35.9	
≥3	194	8.6	329	13.7	
Diabetes	180	8.0	341	14.2	<0.001
Hypertension	597	26.5	928	38.6	<0.001
Death	207	45.5	378	66.8	<0.001
Alive with graft failure^#^	248	54.5	188	33.2	
Crude patient survival rate (%)^$^					
3rd month	99.0		98.5		<0.001
1st year	96.9		95.8		
3rd year	94.0		92.1		
5th year	91.7		87.8		
7th year	88.4		82.2		
9th year	84.9		76.8		
10th year	83.0		73.1		
Crude graft survival rate (%)^$^					
3rd month	97.4		96.9		0.649
1st year	93.4		93.8		
3rd year	87.0		87.7		
5th year	80.1		81.3		
7th year	74.3		73.0		
9th year	67.6		65.7		
10th year	64.3		59.8		

Note: Results are n (%) or means (SD).

CCI = Charlson comorbidity index.

* The chance to reject null hypotheses, assuming no difference in demographic characteristics between patients receiving a kidney transplant in Taiwan and patients receiving a kidney transplant overseas, by using chi-square tests (for categorical data), Student’s *t-*tests (for continuous data), and log-rank tests (for patient and graft survival rate).

^†^ The diagnoses recorded in the National Health Insurance dataset within 1 year (excluding the index hospitalization for the kidney transplant) before receiving a kidney transplant was used to calculate CCI score. Because patients undertaking dialysis defined our study cohort, we excluded the diagnosis of renal failure (defined as at least three outpatient service claims or one single hospitalization) from index calculations.

^#^ the patient resumed dialysis but was still alive.

^$^ January 1, 1998 to June 30, 2009, excluding domestic graft failure and those who died within one month.

As shown in Table 2, overseas LT recipients were older (50.3 vs 43.0 years, p<0.001), mainly adult (97.4% vs 83.5%, p<0.001), and male-dominant (82.0% vs 69.0%, p<0.001). In addition, more of them had hepatocellular carcinoma compared to their domestic counterparts (64.1% vs 39.9%, p<0.001).

**Table 2:** Characteristics of liver transplant recipients in 1999–2009, domestic vs overseas.

	Domestic LT recipients (N = 1658)		Overseas LT recipients (N = 540)		*p*-value*
	n	%	n	%	
Sex (male)	1144	69.0	443	82.0	<0.001
Age at transplantation (years, mean ± SD)	43.0 (19.4)		50.3 (13.2)		<0.001
≤18	273	16.5	14	2.6	<0.001
18–45	567	34.2	218	40.4	
45–60	629	37.9	183	33.9	
>60	189	11.4	125	21.1	
CCI^†^ (mean ± SD)^†^	0.99 (1.36)		1.22 (1.78)		0.006
0	832	50.2	260	48.2	0.164
1–2	598	36.1	189	35.0	
≥3	228	13.7	91	16.9	
Hepatocellular carcinoma	661	39.9	346	64.1	<0.001
Viral hepatitis	1147	69.2	378	70.2	0.720
Indication for liver transplant^†^					
Fulminant failure	102	6.2	9	1.7	<0.001
Biliary atresia	214	12.9	12	2.2	
Biliary cirrhosis, metabolic	69	4.2	6	1.1	
Hepatocellular carcinoma with cirrhosis	647	39.0	307	56.9	
Hepatocellular carcinoma without cirrhosis	14	0.8	39	7.2	
Alcoholic liver cirrhosis	187	11.3	41	7.6	
Viral hepatitis with cirrhosis	399	22.9	102	18.9	
Other	26	1.6	24	4.4	
Crude patient survival rate (%)^$^					
3rd month	95.6		97.0		<0.001
1st year	89.2		79.8		
3rd year	83.9		62.3		
5th year	79.5		54.7		
7th year	77.8		49.9		
9th year	77.3		49.9		
10th year	75.2		49.9		

*TThe chance to reject null hypotheses, assuming no difference in demographic characteristics between patients receiving a kidney transplant in Taiwan and patients receiving a kidney transplant overseas, by using chi-square tests (for categorical data), Student’s *t-*tests (for continuous data), and log-rank tests (for patient and graft survival rate).

^†^ The diagnoses recorded in the National Health Insurance dataset within 1 year (excluding the index hospitalization for the liver transplant) before receiving a liver transplant was used to calculate CCI score and indication of liver transplant. When calculating CCI score, we excluded diagnoses of mild hepatitis, moderate hepatitis, and hepatocellular carcinoma from index calculations. The diagnosis was defined as at least three outpatient service claims or one single hospitalization.

^$^ Excluding domestic patients who died within one month.

### Clinical outcome

**Fig 2:**
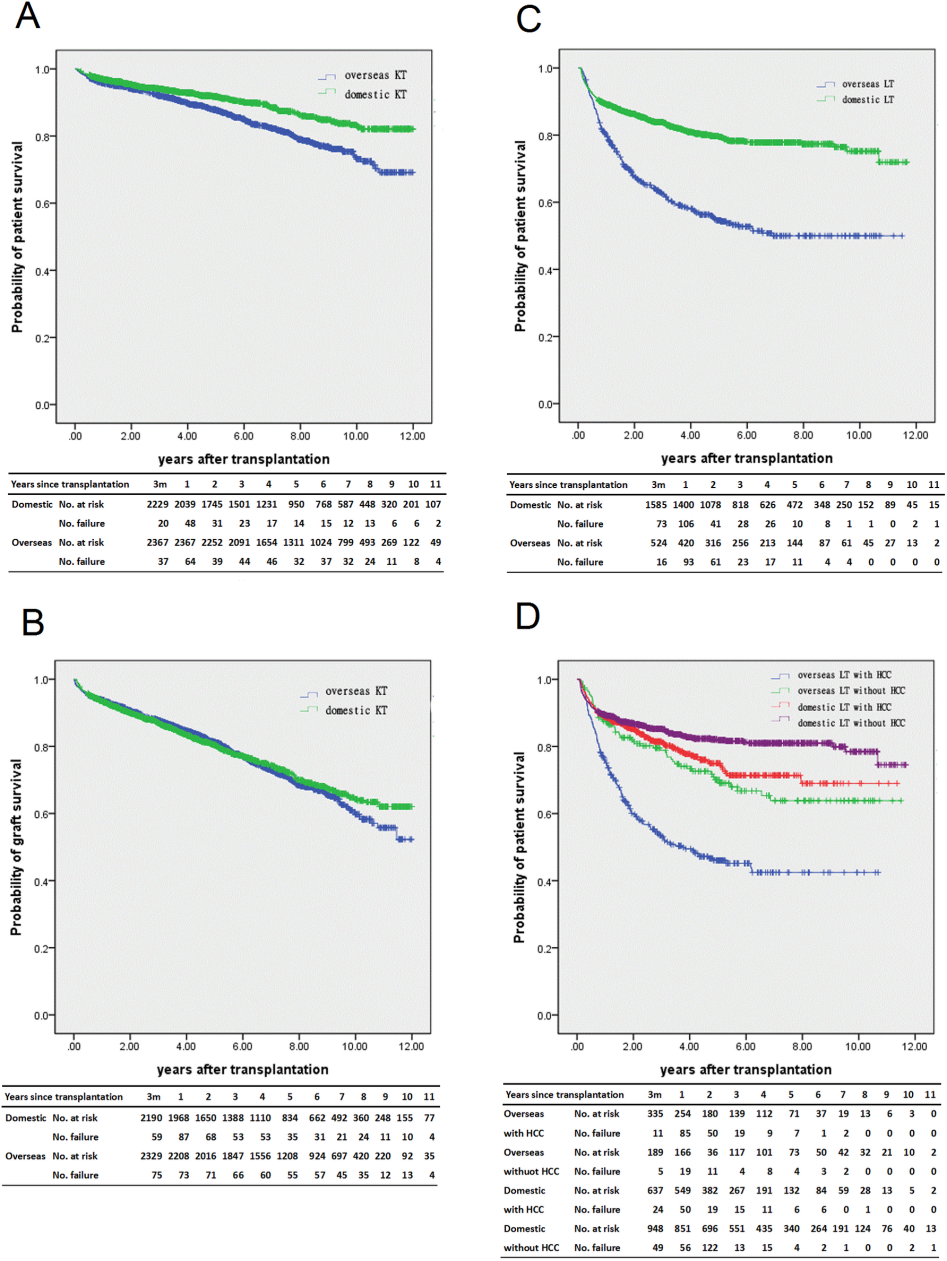
Kaplan–Meier estimates of survival for overseas vs domestic transplant recipients. (A) Patient survival for kidney transplant recipients, log-rank test P < 0.001; (B) graft survival for kidney transplant recipients, log-rank test P = 0.649; (C) patient survival for liver transplant recipients, log-rank test P < 0.001; (D) patient survival for liver transplant recipients, categorized by location and whether the patient had hepatocellular carcinoma, log-rank test P < 0.001.

Table 1 and Fig 2 show the domestic KT recipients had a significantly better crude patient survival rate than the overseas recipients (log rank test p<0.001) but a similar graft survival rate (log rank test p = 0.649). In Cox proportional hazards model (Table 3), the risk factors of mortality for KT recipients were older age (>65 y/o vs ≤35 y/o, aHR = 5.00 [3.39–7.36], p<0.001), male (aHR = 1.35 [1.14–1.61], p<0.001), higher CCI score (≥3 vs 0, aHR = 1.53 [1.20–1.94], p<0.001), and longer pre-transplantation dialysis time (≥1 yr vs no dialysis, aHR = 1.36 [1.01–1.81], p = 0.040). Regarding kidney graft failure, there was no difference between domestic or overseas kidney recipients (aHR = 0.88 [0.77–1.01], p = 0.068), but older age (>65 y/0 vs <35 y/o, aHR = 2.15 [1.57–2.94], p<0.001), male (aHR = 1.15 [1.02–1.31], p = 0.029), higher CCI score (≥3 vs 0, aHR = 1.41 [1.16–1.70], p = 0.001) and longer pre-transplantation dialysis time (>1yr vs no dialysis, aHR = 1.46 [CI 1.16–1.82], p = 0.001) were still considered risk factors for graft failure (Table 3).

**Table 3:** Risks of mortality and graft failure for kidney transplant recipients, January 1, 1998 to June 30, 2009.

	Graft failure HR (95% CI)	*p*-value*	Overall survival HR (95% CI)	*p*-value*
Location				
Domestic	1.0		1.0	
Overseas	0.88 (0.77–1.01)	0.068	1.10 (0.92–1.33)	0.295
Age at transplantation (years)				
<35	1.0		1.0	
35–45	0.97 (0.80–1.18)	0.774	1.20 (0.88–1.63)	0.254
45–55	1.17 (0.97–1.41)	0.094	2.05 (1.54–2.72)	<0.001
55–65	1.60 (1.30–1.97)	<0.001	3.43 (2.55–4.62)	<0.001
≥65	2.15 (1.57–2.94)	<0.001	5.00 (3.39–7.36)	<0.001
Sex				
Female	1.0		1.0	
Male	1.15 (1.02–1.31)	0.029	1.35 (1.14–1.61)	<0.001
CCI				
0	1.0		1.0	
1–2	1.11 (0.96–1.28)	0.147	1.14 (0.95–1.38)	0.161
≥3	1.41 (1.16–1.70)	0.001	1.53 (1.20–1.94)	0.001
HD before KT				
0	1.0		1.0	
<1 year	1.27 (1.01–1.61)	0.044	1.09 (0.80–1.49)	0.576
≥1 year	1.46 (1.16–1.82)	0.001	1.36 (1.01–1.81)	0.040

*Hazard ratio (HR) was used to estimate excess risks of mortality and graft failure for those receiving transplants overseas vs domestically, by using a multivariable Cox proportional hazards regression model adjusted for age, sex, hemodialysis duration prior to transplantation, CCI score, and location of transplantation site.

Regarding liver transplants, the domestic LT recipients had significantly better survival probabilities than those of the overseas LT recipients in the crude rate (log-rank test p<0.001, Table 2 and Fig 2). Overseas LT recipients with prior hepatocellular carcinoma had the lowest survival rate (Fig 2). In Cox proportional hazards models, due to significant interaction between location of transplantation and history of hepatocellular carcinoma (p<0.001), we separated the subjects into two groups according to their history of hepatocellular carcinoma (Table 4). In the hepatocellular carcinoma group, overseas LT had a significantly higher hazard ratio for patient mortality (aHR = 2.65 [2.08–3.38], p<0.001) after adjusting for age, sex, and CCI score. On the other hand, in the non-hepatocellular carcinoma group, the mortality rate of overseas LT was not different from that of domestic LT (aHR = 1.31 [0.94–1.82], p = 0.107). Older age (>60 y/o vs ≤18 y/o, aHR = 2.32 [1.37–3.93], p = 0.001) was another risk factor for patient mortality.

**Table 4:** Risk of mortality for liver transplant recipients, 1999–2009.

	Patient without hepatocellular carcinoma		Patient with hepatocellular carcinoma	
	Overall survival HR (95% CI)	*p*-value*	Overall survival HR (95% CI)	*p*-value*
Location				
Domestic	1.0		1.0	
Overseas	1.31 (0.94–1.82)	0.107	2.65 (2.08–3.38)	<0.001
Sex				
Female	1.0		1.0	
Male	1.04 (0.78–1.39)	0.778	1.00 (0.73–1.38)	0.979
CCI				
0	1.0		1.0	
1–2	0.99 (0.73–1.35)	0.965	1.01 (0.77–1.31)	0.962
3+	1.19 (0.80–1.76)	0.394	1.18 (0.85–1.62)	0.323
Age at transplantation				
0–18	1.0			
18–45	1.43 (0.93–2.18)	0.102	1.0 ^**$**^	
45–60	1.94 (1.26–2.97)	0.002	0.84 (0.65–1.10)	0.207
≥60	2.32 (1.37–3.93)	0.001	0.92 (0.67–1.27)	0.621

* Hazard ratio (HR) was used to estimate excess risks of mortality for those receiving transplants overseas vs domestically, by using a multivariable Cox proportional hazards regression model adjusted for age, sex, CCI score.

^**$**^ 0–45 as reference because sparse data in <18 y/o strata.

We further identified that post-KT malignancy and liver disease were the two main causes of death for overseas KT recipients compared to those for the domestic KT recipients. On the other hand, hepatocellular carcinoma was the major cause of death for overseas LT recipients (69.0 per 1000 person-years; IRR = 6.58 [4.69–9.23], p<0.001) compared to their domestic counterparts (8.2 per 1000 person-years) (Table 5).

**Table 5:** Cause of death in kidney and liver transplantation recipients.

	**Domestic KT recipient**		**Overseas KT recipient**						
Cause of death*		**Incidence per** **1,000 person-year** **(95% CI)**		**Incidence per 1,000 person-year** **(95% CI)**	***p*-value**		***IRR***^#^		***p*-value**
	n	(Total 11017 person-year)	n	(Total 13184 person-year)					
Malignancy&	33	3.0 (2.1–4.2)	105	8.0 (6.6–9.6)	<0.001		1.82 (1.21–2.73)		0.004
Infection	50	4.6 (3.4–6.0)	101	7.7 (6.3–9.3)	0.003		1.07 (0.74–1.53)		0.710
Liver disease	15	1.4 (0.8–2.3)	43	3.2 (2.4–4.4)	0.004		1.78 (0.97–3.27)		0.063
CKD	53	4.8 (3.7–6.3)	71	5.4 (4.3–6.9)	0.534		0.74 (0.49–1.11)		0.147
Others	56	5.1 (3.9–6.6)	58	4.4 (3.4–5.8)	0.441		0.67 (0.45–1.00)		0.050
	**Domestic LT recipient**		**Overseas LT recipient**						
**Cause of death**^†^		**Incidence per** **1,000 person-year** **(95% CI)**		**Incidence per** **1,000 person-year** **(95% CI)**	***p*-value**		***IRR***^#^		***p*-value**
	n	(Total 6104 percent-year)	n	(Total 1854 percent-year)					
Hepatocellular carcinoma	50	8.2 (6.5–10.8)	128	69.0 (52.0–82.1)	<0.001		6.58 (4.69–9.23)		<0.001
Infection	45	7.4 (5.5–9.9)	22	11.9 (7.8–18.0)	0.068		1.27 (0.74–2.21)		0.387
Liver disease	99	16.2 (13.3–19.7)	34	18.3 (13.1–25.7)	0.538		0.94 (0.62–1.42)		0.761
Complication	65	10.6 (8.4–13.6)	27	14.6 (10.0–21.2)	0.172		1.26 (0.77–2.06)		0.349
Others	38	6.2 (4.5–8.6)	18	9.7 (6.1–15.4)	0.121		1.44 (0.80–2.58)		0.226

The causes of mortality were primarily defined as the principal diagnosis when patient expired unless some vague diagnoses for nosology such as cardiovascular symptoms (ICD9 = 785) or other disease of lung (ICD9 = 518). In these cases, we chose the second diagnoses to define the causes of death.

The mortality rate was estimated by cases per 1000 person-years, which were calculated as the time elapsed from the transplantation to the death date, or the end of follow-up, whichever came first. The calculation of a 95% CI for the mortality rate was based on the Poisson distribution.

*Causes of death in kidney transplantation: Liver disease-included liver cirrhosis, acute hepatitis, hepatic failure; chronic kidney failure include -complication after kidney transplantation, chronic kidney failure and kidney transplantation. Others-include cardiovascular and cerebrovascular accident, GI disease (intestinal perforation, pancreatitis, peritonitis) and others.

& Malignancy in overseas vs. domestic: genitourinary malignancy: 39 vs 12, hepatocellular carcinoma: 29 vs 6, others: 37 vs 15.

^†^Cause of death in liver transplantation: liver disease include hepatic failure, and side effect of hepatic failure. Others include tumor except hepatocellular carcinoma, cardiovascular, cerebrovascular accident and GI disease.

^#^IRR (incident rate ratio) was used to assess association between cause of death and the transplant operation sites (overseas vs. domestic) by using Poisson regression model adjusted for age, sex, and CCI score.

## Discussion

### Features of transplant tourism from Taiwan to China

Our results showed that the overseas transplant group had the following characteristics: male predominant, older, having more comorbidities, having a shorter pre-operative dialysis time in kidney transplant, and more hepatocellular carcinoma cases in liver transplant. The outcomes of overseas transplant were inferior to domestic transplants in crude rate. After adjusting for covariates, no difference was noted in overseas and domestic kidney transplant. However, overseas liver transplant is much worse than domestic liver transplant in the hepatocellular carcinoma group.

There were several reasons that created the different characteristics between domestic and overseas transplant. Taiwan is still a relatively paternalistic society, and males commonly play a dominant role in family finance and income disposition. Older people generally have greater financial and social resources; yet they might have more health problems and comorbidities, which put them at a disadvantage in rank on the transplant-waiting lists and may even lead to them being excluded for surgery. Hence, they are more likely to grasp an opportunity for overseas transplantation. The pre-transplantation dialysis period is shorter in the overseas group, which indicates a shorter waiting period and the commercial nature of overseas transplantation. The same reason applies to overseas liver transplants, with more recipients being older and male.

#### Patient and graft survivals

Two previous studies show that the clinical outcomes of overseas KTs were comparable to those of domestic KTs after 2000 [7,14]; however, those studies had brief follow-up periods (< 5 years) and used only one institution with limited case numbers. In our study, the crude patient survival rate was better for domestic KT recipients, but there was no difference in graft survival. The higher mortality rate in overseas KT recipients might have been reduced by a low kidney graft failure rate (overseas vs. domestic: 33.2% vs. 54.5%, Table 1). This is consistent with the general conception that organs procured from executed prisoners (especially young males) in China are similar to organs from living donors, and hence, have better “quality” than the domestic deceased organs which are mainly from brain-dead patients. However, after adjusting for covariates, the mortality rate was similar between domestic and overseas KT patients. The poor survival rate in overseas patients is attributed to the characteristics of overseas patients (old age, more comorbidity, and male).

#### Malignancy

We found that the main causes of death for KT, especially in overseas transplants, were malignancy and liver disease. The most common malignancies in overseas KT recipients were genitourinary malignancy (kidney or bladder cancer) and hepatocellular carcinoma. Tsai et al.[15] also reported a high de novo malignancy rate in renal transplant tourism compared to that of domestic renal transplant recipients. The 10-year cumulative cancer incidence of the tourism group (21.5%) was significantly higher than that of the domestic group (6.8%), and the most common cancers were urothelium carcinoma and hepatocellular carcinoma. The high cancer incidence in the tourism group might be related to older age, more depleting antibody induction therapy, and omitted pre-transplant cancer screening procedures.

In liver transplants, patient survival was remarkably worse among overseas recipients. Overseas hepatocellular carcinoma patients had the worst prognosis compared with other groups. In Taiwan, to ensure standard quality, the NHI program reimburses live LT according to UCSF criteria [16] and cadaver LT according to Milan criteria [17]. In overseas LT, 64.1% had hepatocellular carcinoma before operation compared to 39.9% in domestic groups; but the hepatocellular carcinoma mortality rate was 69.0 compared to 8.2 per 1000 person-years (p<0.001), which implies that most overseas LT recipients were not suitable candidates for LT and inevitably had a high hepatocellular carcinoma recurrence rate and high mortality. Similar to our findings, Allam et al. also reported poor outcomes for LT patients who received transplantation in China, showing one- and three-year cumulative patient survival rates of 83% and 62%, respectively, compared to 92% and 84% in domestic hospitals [18]. The main reason for this discrepancy may be less prudent selection criteria for transplantation in China because 41 (55%) of the patients who received overseas transplantation had been denied liver transplantation at domestic hospitals due to multiple comorbidities, exceeding the age limit, or advanced hepatocellular carcinoma. In other words, some LT cases might not be medically indicated and some KT cases were clinically suboptimal for transplantation in the overseas groups, which might contribute to the poorer outcome of the overseas groups.

### Transplant tourism in Asia and organs from executed prisoners

Surveys show that a remarkable number of people from many Asian countries in addition to Taiwan also traveled to China for transplantation: there were 462 KT and 504 LT cases from South Korea to China between 2001 and 2006 (19); 801 cases of KTs from Malaysia to China, which accounted for half of the country’s total KTs between 2002 and 2011 (20); and 752 cases of KT from Israel to the Philippines and China between 2001 and 2007 (21). Saudi Arabia also had 650 overseas KT, though not specifically mentioning which countries they went to (22). [19–22]. Although China is reforming its transplantation policy and has announced that it is no longer using organs from executed prisoners, critics have continued to question whether this practice has remained [23]. The WMA Statement on Organ and Tissue Donation (2012) indicates that “in jurisdictions where the death penalty is practised, executed prisoners must not be considered as organ and/or tissue donors. While there may be individual cases where prisoners are acting voluntarily and free from pressure, it is impossible to put in place adequate safeguards to protect against coercion in all cases” [24]. In relation to the commercial aspects of transplant tourism, the Declaration of Istanbul contains prohibitions against a range of practices, including “a ban on all types of advertising (including electronic and print media), soliciting, or brokering for the purpose of transplant commercialism, organ trafficking, or transplant tourism.” And the recently adopted “Convention against Trafficking in Human Organs” (2014) by the Council of Europe obligates ratifying states to criminalize ^“^trafficking in human organs” [25].

#### Taiwan and transplant tourism trends

Overseas transplants increased rapidly after 2000, perhaps due to improved surgical techniques and transplantation outcomes, as well as to increased organ supply and brokering activity in China. In 2006, China admitted using organs from executed prisoners [10]—a practice prohibited by international professional societies and condemned by human rights groups. As the public, the media, and NGOs began to better understand the unethical nature of transplant tourism, pressure started to grow in Taiwan. While international organizations were exercising pressure on China and requesting legal reform on transplantation policy, Taiwan’s government prohibited medical personnel from involvement in any form of organ brokering. China passed its Human Organ Transplant Act in 2007. Since these policy changes, the number of transplant tourists from Taiwan to China has decreased remarkably. This might be due to an increased awareness of related ethical/legal controversies, but also due to the escalated expense of organ trafficking resulting from outlawing the organ trade, which led to reduced availability of organs (prices nearly doubled and even tripled according to the authors’ local survey).

In June 2015, Taiwan passed amendments to the Human Organ Transplantation Act. Organ brokers and patients receiving illegal organ transplants no matter domestically or overseas could face a maximum of five years imprisonment and a fine of up to USD 50,000. Criminalizing “patients” for illegal transplantation was disputed during the law amendment discussions (2013–2015). Some transplantation professionals, patient groups and Ministry of Health & Welfare expressed sympathy for patients who receive such transplantation and raised opposing opinion because patients might be desperate and hopeless while so doing. Yet the Ministry of Justice and human right organization supported such amendment based on the principle that human rights protection and punishment should be applied equally to brokers and buyers in an illegal organ trade [26,27]. After the Act passed, compulsory registration for overseas transplantation is required, which will promote transparency in transplant tourism and therefore may serve as a deterrent. Prohibiting using executed prisoners as organ donors to follow international guideline was implemented. Increased domestic organ donation strategies including “mandatory choice” and “required request” in deceased organ procurement policy, promoting “donation after circulatory death,” and allowing “paired exchange” were all included in the amended law [28]. Although it will take time to observe the amendments’ actual effects on transplant tourism, the overall trend has shown a reduction in numbers.

### Policy suggestion for regulating transplant tourism in Asia

International guidelines concerning organ transplantation all call for adoption of a paradigm that involves governments taking national-level responsibility for fulfilling patients’ needs for organ donation and transplantation, and for ending unethical/illegal organ trafficking and commercialization [29]. In 2007, the Philippines prohibited foreigners from travelling to that country for transplantation, which quickly led to a remarkable decrease in such cases [30]. In 2008, Israel passed a law banning the sale, purchase, and brokerage of organs, both in Israel and abroad; and it has arrested brokers. As a result, transplant tourism to China from there seems to have ceased [31]. Despite these regulatory efforts in reducing organ tourism, the issue remains a complex, conflicting, ethical/legal challenge in many Asian countries. Politicians, patients, doctors, brokers, and other stakeholders have engaged in a power struggle to protect their respective interests, which in turn has made ethical and effective legislation difficult to accomplish. Comprehensive and enforceable national and international regulatory frameworks within Asian regions, which could be similar to the Convention against Trafficking in Human Organs (2014) by the Council of Europe, are indeed needed yet lacking. The WHO Guiding Principles on Human Cell, Tissue and Organ Transplantation (2010)—requiring relevant transplantation information to be open, accessible, and monitored—could serve as a reference, with enacting principles 10 and 11 (“traceability” and “transparency,” respectively) serving as the first step [32].

We therefore propose that Asian countries, as well as other countries involved with transplant tourism, adopt practical strategies and legislation so as to effectively reduce transplant tourism and organ trafficking. For example, they should:

1. Set up compulsory registration policies for overseas transplantation for monitoring this practice.
2. Sanction and punish all parties involved in organ trade and brokering.
3. Develop international and national legislation to criminalize and prevent all activities involving organ trafficking.
4. Develop an effective national organ procurement and donation policy so as to reduce the organ shortage and achieve national self-sufficiency in transplant organs.
5. Continue efforts to stop the use of organs from executed prisoners in China.

### Strengths and limitations of this research

A strength of our study is that all the overseas transplant patients were identified and the results can be generalized. Additionally, the cohort is larger and with longer follow-up times (an 11-year cohort) than previous studies on transplant tourism (7,18). However, this study has several limitations. This is a retrospective study and recruited only overseas transplantation patients who survived, returned to Taiwan, and received anti-rejection therapies. Early intra-hospital mortality cases, in which the patients died after transplantation and failed to return to Taiwan, were not available in our research. This problem is common to all similar studies investigating the outcome of transplant tourism. To avoid overestimating the outcome of overseas transplants, we excluded domestic recipients who died within one month or who resumed dialysis within one month after the transplant operation to make the overseas and domestic groups more comparable. In addition to donor quality, some key variables in the LT models that had affected post-LT survival were not available, including various aspects of donor quality, pre-operative laboratory data, and the characteristics of hepatocellular carcinoma. Therefore, these confounding factors were not exclusively adjusted in the multivariable models. Due to the unethical nature of the transplant tourism sector, overseas transplant patients usually lack such clinical data and return home with only limited medical information, which hinders fair comparison and comprehensive research. However, the purpose of our study is not to find all the covariates affecting post-transplant survival in order to improve overseas transplants; rather, our study seeks to provide a picture of overseas transplants (trends, patient characteristics, and outcomes) in transplant tourism over the past decade in order to propose possible solutions to this important global health issue.

## Conclusion

Our study gives a basic overview and describes problems of transplant tourism from Taiwan to China. The overseas transplant group had different demographic and clinical compositions than those of the domestic one; hence, the overseas group’s outcome is inferior. Although transplant tourism has decreased after the increased ethical awareness and establishment of relevant professional guidelines and policies, it still exists in many countries. We have reflected upon the ethical controversies of transplant tourism and proposed strategies for policy and legal reform based on recent professional and governmental efforts, as well as developments in Taiwan; these could be useful references for other Asian countries.

## Supporting information

S1 Table Numbers of domestic kidney and liver transplants, stratified by living and deceased status, April 2005–2015. (PDF)

## Abbreviations

KT: kidney transplant
LT: liver transplant
TORSC: Taiwan organ registry and sharing center
NHI: national health insurance
NHIRD: NHI Research Database
SD: standard deviation
CCI: Charlson comorbidity index
HR: hazard ratio
IRR: incidence rate ratio
