# Supplementary material for: The outcomes and controversies of transplant tourism—Lessons of an 11-year retrospective cohort study from Taiwan
Source: PLoS One. 2017 Jun 2;12(6):e0178569. doi: 10.1371/journal.pone.0178569 (PMC5456093; doi:10.1371/journal.pone.0178569)
Supplement: S1 Table — (PDF) [file pone.0178569.s001.pdf]

S1 Table. Numbers of domestic kidney and liver transplants, stratified by living and deceased status, April 2005–2015.

	2005	2006	2007	2008	2009	2010	2011	2012	2013	2014	2015
kidney transplant											
deceased	163	202	177	200	226	190	244	190	197	208	201
living			68	88	90	97	84	73	128	128	103
liver transplant											
deceased	61	72	75	83	89	91	107	94	97	104	108
living			187	238	266	344	401	431	447	484	505

1. The data were from TORSC (Taiwan Organ Sharing Center)

2. TORSC was established in 2003 and started to distribute, under an internal regulation, the transplants from the deceased donors to the needed since April, 2005. TORSC began to collect living donor data since 2007 by asking the hospitals that were involved in organ transplantation to report the numbers and outcomes of the domestic transplants twice a year. Therefore, in S1 Table, the number of deceased donor was available since 2005 and more reliable after 2007; the number of living donor was available since 2007. The data in the TORSC rely on hospitals' reports, so they are not completely comparable with the data shown in Figure 1, which were derived from the NHIRD database.
